# Crystal structure of *Trichinella spiralis* calreticulin and the structural basis of its complement evasion mechanism involving C1q

**DOI:** 10.3389/fimmu.2024.1404752

**Published:** 2024-04-16

**Authors:** Zhihui Jia, Wen Yu, Jingmo Li, Mingming Zhang, Bin Zhan, Liming Yan, Zhenhua Ming, Yuli Cheng, Xiaolin Tian, Shuai Shao, Jingjing Huang, Xinping Zhu

**Affiliations:** ^1^ Department of Medical Microbiology and Parasitology, School of Basic Medical Sciences, Capital Medical University, Beijing, China; ^2^ Departments of Pediatrics and Molecular Virology and Microbiology, National School of Tropical Medicine, Baylor College of Medicine, Houston, TX, United States; ^3^ Ministry of Education Key Laboratory of Protein Science, School of Medicine, Tsinghua University, Beijing, China; ^4^ College of Life Science and Technology, Guangxi University, Nanning, China; ^5^ Ministry of Education Key Laboratory of Bioinformatics, School of Life Sciences, Tsinghua University, Beijing, China; ^6^ Beijing institute of Clinical Medicine, Capital Medical University Affiliated Beijing Friendship Hospital, Beijing, China

**Keywords:** *Trichinella spiralis* calreticulin, x-ray crystallography structure, immune evasion, C1q, peptide modulator

## Abstract

Helminths produce calreticulin (CRT) to immunomodulate the host immune system as a survival strategy. However, the structure of helminth-derived CRT and the structural basis of the immune evasion process remains unclarified. Previous study found that the tissue-dwelling helminth *Trichinella spiralis* produces calreticulin (TsCRT), which binds C1q to inhibit activation of the complement classical pathway. Here, we used x-ray crystallography to resolve the structure of truncated TsCRT (TsCRT^Δ^), the first structure of helminth-derived CRT. TsCRT^Δ^ was observed to share the same binding region on C1q with IgG based on the structure and molecular docking, which explains the inhibitory effect of TsCRT on C1q-IgG–initiated classical complement activation. Based on the key residues in TsCRT^Δ^ involved in the binding activity to C1q, a 24 amino acid peptide called P^TsCRT^ was constructed that displayed strong C1q-binding activity and inhibited C1q-IgG–initiated classical complement activation. This study is the first to elucidate the structural basis of the role of TsCRT in immune evasion, providing an approach to develop helminth-derived bifunctional peptides as vaccine target to prevent parasite infections or as a therapeutic agent to treat complement-related autoimmune diseases.

## Introduction

As a bridge between the innate and adaptive immune response, the human complement system consists of more than 50 protein components and serves as a first line of defense against infection by pathogens including bacteria, fungi, and parasites. It can be activated through the classical, lectin, or alternative pathways, all of which eventually converge in the formation of the membrane attack complex (MAC), complementing the ability of antibodies and other host immune molecules to kill the invading pathogens (1, 2). Of the three pathways, the classical pathway plays the most important role in the defense against foreign pathogens and is initiated by binding of antigen–antibody (IgG/IgM) complexes to the core initiator C1q.

Of all the infective pathogens, parasites account for a large proportion. Protozoa and helminths have both developed sophisticated strategies to evade immune attack by manipulating the host immune system during the long revolutionary process of parasite-host interplay. One of these strategies is to secrete proteins that target complement components to inhibit MAC formation (2–4). Calreticulin (CRT), for instance, is a key protein in immune evasion against complement attack. It has a high affinity for Ca^2+^ and is located mainly in the endoplasmic reticulum in all mammalian cells except for erythrocytes. With a relatively high conservation in terms of both the sequence and structure, CRT has multiple functions including as a chaperone and apoptosis adaptor and in antigen presentation (5, 6). According to the predicted structural model, CRT consists of a globular lectin domain including both the N- and C-termini and a long arm-like domain in the middle known as the P (Proline-rich) domain. The inhibitory effects of parasite-secreted CRT against host complement activation have been identified in both parasitic protozoa and helminths (7). *Trypanosoma cruzi* (*T. cruzi*)–produced calreticulin (TcCRT) was able to inhibit the C1q-mediated hemolysis *in vitro* and is possibly involved in the defense against host complement attack (8, 9). Meanwhile, TcCRT may play an important role in invading host macrophages by binding to C1q on the surface of trypomastigotes, which triggers phagocytosis (10, 11). *Entamoeba histolytica* (*E. histolytica*) trophozoite–produced calreticulin (EhCRT) plays a similar role in the complement-involved opsonization and phagocytosis of erythrocytes and apoptotic lymphocytes (12–14). In addition to protozoa, helminths also produce CRT proteins that have a similar inhibitory effect on the activation of host complement. *Trichinella spiralis* (*T. spiralis*)–produced calreticulin (TsCRT), for instance, is capable of binding host complement C1q to hamper the initiation of the classical complement pathway, as a survival strategy (15, 16). CRT originating from *Necator americanus* (*N. americanus*), *Haemonchus contortus* (*H. contortus*), *Brugia malayi* (*B. malayi*), and *Opisthorchis viverrini* (*O. viverrini*) also inhibit C1q-initiated complement activation in a dose-dependent way to facilitate the survival of parasites in hosts (7, 17–21).

It is apparent that parasitic pathogens have evolutionarily developed a complement evasion strategy involving the production of CRT to invade and establish infection within hosts. However, the interaction between CRT and C1q and the inhibitory mechanism underlying complement activation is not known, especially at the structural level.

Mammalian-derived C1q is a 410 kDa homohexamer composed of 18 polypeptide chains clustered in six heterotrimeric modules; each of the modules comprises an N-terminal collagen-like fragment (CLF) and a C-terminal globular region (GR) (22, 23). Within their immune evasion role, CRT proteins derived from different parasites may adopt different C1q-binding modes. Protozoal *T. cruzi*-produced CRT (TcCRT) was reported to interfere with the activation of the complement classical pathway through binding to C1q collagen-like fragment (C1q-CLF) (4, 11, 24). Meanwhile, nematode *B. malayi*–produced calreticulin (BmCRT) and TsCRT were predicted to interact with C1q-GR to interfere with the initiation of complement activation (16, 20). Therefore, it is reasonable to hypothesize that portions with various conformations must be remained in CRT derived from different parasites to perform different binding modes toward C1q although there existing a relatively high conservation among the structure of CRT. However, most of these findings are based on biochemical functional experiments or virtual molecular docking using predicted CRT structural models, little has been done to explore the molecular interaction between CRT and complement based on actual structural analysis until now.

So far, the structures of the globular domain of CRT from *Homo sapiens* (*H. sapiens*) and *Mus musculus* (*M. musculus*), as well as those of two protozoan CRT proteins TcCRT and EhCRT, have been determined using x-ray crystallography—all of the individual CRT structure are P domain–truncated versions (25–30). No helminth-derived CRT structures have been resolved yet. In this study, we successfully resolved the structure of P-domain–truncated TsCRT—the first helminth CRT structure to date—providing a real helminth CRT structure to interpret and explore the molecular mechanism underlying CRT-involved complement evasion through interaction with C1q. The accordingly derived C1q-binding peptide P^TsCRT^ was constructed, and its potential to modulate the complement classical pathway was functionally evaluated preliminarily.

## Materials and methods

### Constructs and expression of different fragments of TsCRT

To screen for the construct of TsCRT that retains C1q-binding activity and can be crystalized for structural analysis, the full-length TsCRT and truncated TsCRT (TsCRT^Δ^) consisting of the C-terminus globular domain (22–209) and N-terminal globular domain (305–364) with the P domain (210–304) replaced with a GSG linker. The two constructs were cloned into the bacterial expression vector pET28a(+) using BamHI and XhoI sites. The primers used for cloning are listed in **Supplementary Table S1**. The residuals with potential binding ability to C1q were mutated using MultiS Fast Mutagenesis Kit V2 (Vazyme Technology, Nanjing, China) to determine their actual involvement in the functional binding to C1q. The correct sequences of the final constructs were verified by double-stranded DNA sequencing.

The recombinant plasmid DNAs with correct sequences were transformed into Rosetta (DE3) cells (Tiangen, Beijing, China), and the recombinant proteins were expressed under induction of 0.1 mM IPTG for 16h–20h at 16°C. The bacterial cells were disrupted in 50 mM Tris, 300 mM NaCl, 2 mM CaCl_2_, and 10% glycerol (pH 8.0) with ultrasonication. The expressed soluble recombinant proteins in the lysate supernatant were purified with Ni^2+^ affinity chromatography purification (GE Healthcare LIfe Sciences, California, USA) and polished with a Superdex75 10/300 prep grad gel filtration column. The final concentration of purified recombinant proteins was 10 mg/ml in buffer 20 mM tris, 150 mM NaCl, 5 mM CaCl_2_, pH 8.0. The purity and identity of the purified recombinant proteins were analyzed with sodium dodecyl-sulfate polyacrylamide gel electrophoresis (SDS-PAGE), Western blot using mouse anti-His antibody.

### Crystallization

Initial crystallization conditions were screened utilizing a hanging-drop vapor diffusion method with commercial kits (Hampton Research, California, USA). Needle-like crystals were obtained by mixing 1 µl of TsCRT^Δ^ (total 10 µg) and 1 µl of reservoir solution (0.2 M ammonium acetate, 30% w/v polyethylene glycol 4000, 0.1 M sodium citrate tribasic dihydrate, pH 5.6) for 5–7 days at 18°C. For cryoprotection, 10% glycerol was added to the reservoir composition.

### X-ray structure diffraction data collection, structure determination, and refinement

For data collection, crystals were picked up in a nylon loop and flash-cooled in liquid nitrogen. All diffraction data were collected on beamline BL19U at the Shanghai Synchrotron Radiation Facility (SSRF). The data were integrated and scaled with *XDS* (a data processing package for structure determination) (31). The initial density map and structural model were calculated in Phaser-MR of PHENIX using the predicted structural model of TsCRT with AlphaFold2 for molecular replacement (32). The final model was manually built in *Coot* (a model building program for structure determination) (33) and further refined in PHENIX. The data collection and refinement statistics are listed in **Tables 1** and **2**. The structure of TsCRT^Δ^ was refined to 2.76 Å resolution. The website for AlphaFold2 is as follows: https://colab.research.google.com/github/sokrypton/ColabFold/blob/main/AlphaFold2.ipynb.

**Table 1:** TsCRT^Δ^ data collection statistics.

Parameters	TsCRT^Δ^
Cell parameters	
a (Å)	83.52
b (Å)	154.07
c (Å)	100.78
α, β, γ (°)	90.00, 108.32, 90.00
Space group	P12_1_1
Wavelength used (Å)	0.9792
Resolution (Å)	81.28–2.91 (2.76–2.76)^b^
No. of all reflections	259,814 (41,719)
No. of unique reflections	60,804 (8,925)
Completeness (%)	98.3 (98.8)
Average I/σ(I)	9.4 (2.5)
R_merge_^a^ (%)	13.3 (48.7)

^a^ *R*_merge_ =Σ*_h_* Σ*_l_* |*I_ih_* − *I_h_* |/Σ*_h_* Σ*_Ih_* where *I_h_* is the mean of observations *I_ih_* of reflection *h*.

^b^Numbers in parentheses are corresponding values for the highest resolution shell.

**Table 2:** TsCRT^Δ^ refinement statistics.

Refinement statistics	TsCRT^Δ^
No. of reflections used (σ(F) > 0)	60,766 (6,140)^b^
*R_work_* ^a^ (%)	19.40 (27.66)
*R_free_* ^a^ (%)	24.25 (34.35)
r.m.s.d. bond distance (Å)	0.011
r.m.s.d. bond angle (°)	1.59
Average *B* value (Å^2^)	34.88
No. of protein atoms	16488
No. of solvent atoms	397
Ramachandran plot	
Res. In favored regions (%)	93.78
Res. In allowed regions (%)	5.92
Res. In outlier regions (%)	0.30

^a^ *R*_work_ = Σ(||*F_p_* (obs)| − |*F_p_* (calc)||)/Σ|*F_p_* (obs)|. *R*_free_ is an *R* factor for a pre-selected subset (5%) of reflections that was excluded in the refinement.

^b^Numbers in parentheses are corresponding values for the highest resolution shell.

### Hemolysis assay

To determine the inhibitory effect of TsCRT^Δ^ on C1q-mediated hemolysis, C1q (1 µg) was incubated with different concentrations of TsCRT^Δ^ (0.1 µM, 0.2 µM, 0.4 µM, 0.8 µM, 1.6 µM, and 3.2 µM) or bovine serum albumin (BSA) (3.2 µM), followed by addition of C1q-depleted sera (C1q-D, 1:50) in veronal buffer (Coolaber, Beijing, China) for 1h at 37°C. Fresh sheep red blood cells (5 × 10^6^ cells/ml in veronal buffer) were sensitized with rabbit anti-SRBC antibody (Coolaber, Beijing, China) at 37°C for 30 min; then, cells were added into the TsCRT^Δ^-C1q reaction complex and the incubation was continued for 1h. Hemolysis was quenched using veronal buffer. The mixture was then centrifuged at 1,500 × g for 10 min. The OD_412_ value of hemoglobin in the supernatant was measured. Hemolytic activity was calculated as a percentage compared with the complete hemolysis with water (16).

### Microscale thermophoresis

The Microscale thermophoresis (MST) technique, which is based on the thermophoresis of molecules, provides information about molecule size, charge, and hydration shell for the analysis of protein–protein interactions (34). TsCRT^Δ^ and its mutants were expressed and purified as above. C1q (Merk, Darmstadt, Germany) was fluorescently labeled with a MO-L011 Monolith™ Protein Labelling Kit RED-NHS (NanoTemper, Munich, Germany) according to the manufacturer’s instructions. Labeled C1q (40 nM) was mixed with gradient-diluted TsCRT^Δ^ (highest concentration of 295 µM) or mutants (highest concentrations of H46A/K47A/D49A, D129A, M135A/I151A, Y132A/N158A, D139A/H149A, I140A/G142A, C141A/K145A, P143A/E144A, and W323A/V325A are 73.4 µM, 172 µM, 203 µM, 31.3 µM, 26.6 µM, 265 µM, 250 µM, 297 µM, and 50 µM, respectively) in buffer (50 mM Hepes, 150 mM NaCl, 2 mM CaCl_2_, 0.05% Tween 20, pH 7.4). The mixtures were loaded into silica capillaries K022 (NanoTemper, Munich, Germany) and measured with a Monolith NT.115 system at a constant LED power of 20% and MST power of 50%. The initial data were analyzed by MO. Affinity Analysis software.

To investigate the interference of TsCRT^Δ^/mutants/P^TsCRT^ with the IgG-C1q interaction, competitive binding of IgG and TsCRT^Δ^ to C1q was performed using MST (35); 40 nM C1q (labeled) and 66.7 µM IgG were incubated in the buffer (50 mM Hepes, 150 mM NaCl, 2 mM CaCl_2_, 0.05% Tween 20, pH 7.4) for 15 min to promote protein interaction to achieve balance. Gradient-diluted TsCRT^Δ^ (highest concentration of 20 µM) or mutants or P^TsCRT^ were then added to the IgG-C1q mixture prior to the measurement with LED power of 60% and MST power of 50%. TsCRT^Δ^ or mutants were included to test its inhibitory effect on the affinity between C1q and IgG.

### Hydrogen-deuterium exchange mass spectrometry

Through monitoring of the proton-exchange capacity of the amide skeleton, Hydrogen-deuterium exchange mass spectrometry (HDX-MS) enables mapping of interaction sites (possible peptides involved in interaction) between proteins (36). ​When the protein–protein complex is placed in a buffer with or without deuterium water for a certain duration, the rate of deuterium exchange at the sites involved in the interaction changes in the complex relative to the monomeric protein. High-performance liquid chromatography was used to separate the peptides obtained by enzyme digestion. Finally, based on the mass difference between hydrogen and deuterium, MS was used to monitor the change in hydrogen-deuterium exchange by measuring the peptide center-of-mass shift. ​ The specific procedure was as follows:

For the HDX-MS experiment, 135 µl of 6 mg/ml TsCRT^Δ^/2 mg/ml C1q alone and in the presence of the corresponding ligand were prepared. The buffer, whose pH was 8.0, contained 50 mM Hepes, 150 mM NaCl, and 2 mM CaCl_2_. To initiate deuterium labeling, 5 μL of each 180 μM protein solution was diluted with 45 μL of labeling buffer (20 mM Tris, 500 mM (NH_4_)_2_SO_4_, 99% D_2_O, pH 8.5) at 25°C for 30 s, 60 s, 90 s, and 300 s, and 50 μL of ice-cold quenching buffer (4 M guanidine hydrochloride, 200 mM citric acid, and 500 mM TECP in water solution at pH 1.8, 100% H_2_O) was added to quench the labeling. The reaction tube was then put on ice. Next, 5 μL of 1 μM pepsin solution was added for digestion. At 2 min, the centrifugated sample was placed into the auto-sampler of the Ultimate 3000 HPLC (Thermo Fisher Scienitfic, Waltham, MA USA) for injection. Then, 50 μL of sample was loaded onto and separated by a ACQUITY UPLC 1.7 μm BEH C18 2.1*50 mm column (Waters, Arnhem, Netherlands). Mobile phase A consisted of 1% v/v formic acid, and mobile phase B consisted of 100% acetonitrile and 1% formic acid. The polypeptide was separated by gradient elution at a flow rate of 115 µl/min for 20 min. Mass spectrometry analysis was performed on a Q Exactive Orbitrap mass spectrometer (Thermo Fisher Scientific, Waltham, MA USA). The deuterium exchange levels were determined by subtracting the centroid mass of undeuterated peptide from the centroid mass of deuterated peptide using HDExaminer (Version PD1.4, Thermo Fisher Scientific, Waltham, MA USA).

### Molecular docking

The complex structure of TsCRT with C1q has not been resolved to date. AlphaFold2 is an artificial intelligence tool used for protein structure prediction, and its docking function is a new functionality in the AlphaFold2 version (37). The amino acid sequences of TsCRT^Δ^ and C1q were uploaded, and the one with the highest score (plDDT>85) was picked out as the optimal complex model. The residues involved in the protein–protein interface were analyzed using Chimera software. The website for molecular docking by AlphaFold2 is as follows: https://colab.research.google.com/github/sokrypton/ColabFold/blob/main/AlphaFold2.ipynb.

### Competition binding assay

The potential C1q binding peptide P^TsCRT^ (amino acid sequence: DLEDFNSDTPYRIMFGPDICGPEKR) derived from TsCRT^Δ^ was synthesized and its ability to competitively inhibit C1q binding to IgG was investigated using enzyme-linked immunosorbent assay (ELISA). First, 96-well plates were coated with human IgG (3 µg/ml, Abcam, UK) or the same amount of BSA as control in 100 µl/well of coating buffer overnight at 4°C. Wells were washed with PBST (136.75 mM NaCl, 2.68 mM KCl, 30.25 mM NaH2PO4, 1.76 mM KH2PO4, 0.05% Tween 20, pH 7.4) 3 times and then blocked with 200 µl 3% BSA in PBS at 37°C for 1h. Then, 100 µL of C1q (1 µg) pre-incubated with different doses of TsCRT^Δ^ (0, 4, 8, 16, 32, and 64 µg) was added to each well and incubated for 2h at 37°C. After washing the wells, anti-C1qA mAb (1:10,000, Abcam, Cambridge, UK) and horseradish peroxidase–conjugated goat anti-mouse IgG (1:10,000, ZSGB-BIO, Beijing, China) was sequentially added to the wells followed by incubation for 1h at 37°C. Next, TMB was added (100 µL/well, BD ebioscience, California, USA) as substrate and the absorbance value at 450 nm was measured with an ELISA reader (Thermo Fisher Scientific, Waltham, MA USA).

### Negative staining electron microscopy

C1q was diluted from 1 mg/mL to 0.002 mg/mL for transmission electron microscopy (TEM). Then, 3 μL samples of 0.002 mg/mL C1q were dropped on to a glow-discharged 300 mesh copper grid coated with a thin carbon support film (Zhongjingkeyi Technology, Beijing, China). After standing for 1 min, the samples were blot dried with filter paper. Uranyl acetate stain (2%, w/v) was dropped onto the copper grid to form droplets that completely covered the surface. After standing for 10 consecutive seconds, we blot dried the samples with filter paper and left them to air dry for TEM. The grid was imaged in an FEI Tecnai Spirit D1319 electron microscope operated at 200 kV with a magnification of 67,000×. Images were recorded using a Gatan K2 electron detector (Gatan company) (38).

## Results

### Crystallization and overall structure of TsCRT^Δ^

**Figure 1:**
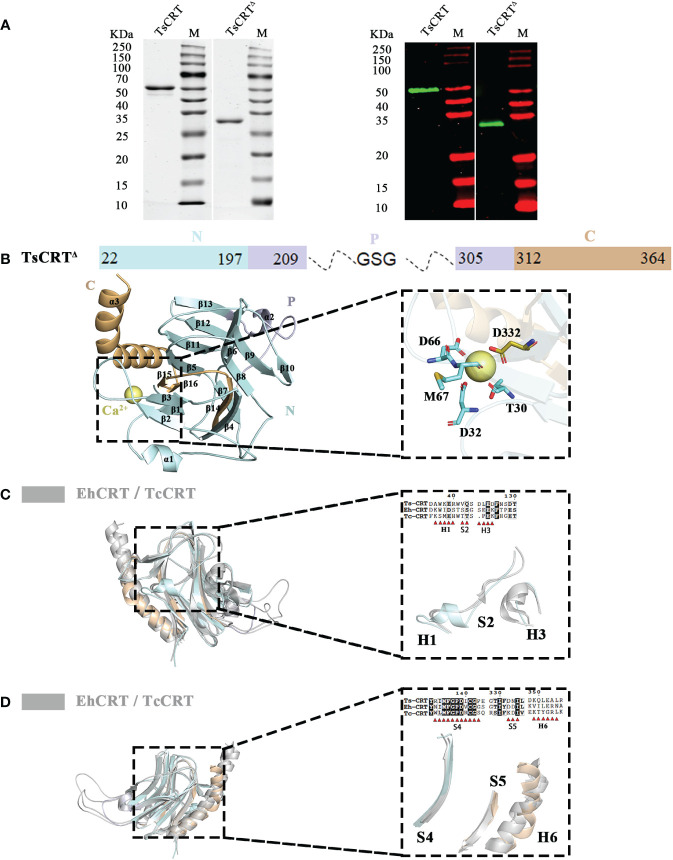
Crystal structure of TsCRT^Δ^ and its alignment with TcCRT & EhCRT. **(A)** 12% SDS-PAGE (left) and Western Blot (right) with anti-His antibody for the purified recombinant TsCRT and TsCRT^Δ^. **(B)** The schematic diagram of TsCRT^Δ^ amino acid sequence is shown on the top, including the N-terminal domain (pale cyan), P domain (blue white), and C-terminal domain (light orange). The P domain and part of C-terminal domain are truncated and replaced with a GSG linker. The overall structure of TsCRT^Δ^ (left) and the magnified details of the Ca^2+^ ion chelating structure coordinated with five residues of TsCRT^Δ^ (T30, D32, D66, M67, and D332) (right) are shown at the bottom. **(C, D)** Alignment of TsCRT^Δ^ with TcCRT and EhCRT revealed six regions with differences in both sequence (39) and structure, namely, H1, S2, H3 **(C)**, S4, S5, and H6 **(D)**. Overall three-dimensional superposition of different calreticulin (left) and the magnified details of six regions of different structures with amino acid sequence alignment (right) are shown. EhCRT (gray, PDB ID: 5HCA), *Entamoeba histolytica* calreticulin; TcCRT (gray, PDB ID: 5HCF), *Trypanosoma cruzi* calreticulin; TsCRT^Δ^ (any colors other than gray), *Trichinella spiralis* calreticulin.

To resolve the structure of TsCRT, various fragments of TsCRT were constructed to test their crystallization capacity. The final construct of TsCRT with capacity to undergo crystallization, called TsCRT^△^, was obtained by truncating the major fragment of the P domain (Residues 209–304) and part of C-terminal globular domain (Residues 365–413), bridged with a GSG linker (**Figure 1B**). The reason to remove the major part of the P-domain was that this flexible arm-like structure in the middle (P-domain) interfered with the crystallization at the current condition.

The correct molecular weight of the expressed recombinant TsCRT^△^ (32 kDa) was confirmed by SDS-PAGE relative to the full-length TsCRT (52 kDa). Both recombinant TsCRT and TsCRT^△^ were recognized by mouse anti-His antibody, indicating their successful expression with His-tag expressed at the N terminus, as expected (**Figure 1A**).

The crystallization of TsCRT^△^ was successfully performed in the pool solution of 0.2 M ammonium acetate, 30% w/v polyethylene glycol 4000, and 0.1 M sodium citrate tribasic dihydrate, pH 5.6 (Hampton Research screening kit). The diffraction data of the crystal was collected and obtained at SSRF for structural determination (**Supplementary Figure S1**).

The structure of TsCRT was determined at a resolution of 2.76 Å. The overall structure of TsCRT^△^ is composed of 3 α helices(α1–α3), 16 β sheets(β1–β16), and several loops between them, in which 2 α helices and 16 β sheets form a β barrel (the globular domain), followed by part of the C-terminus helix(α3) (**Figure 1B**). In addition, a calcium ion, as postulated by analogy with HsCRT(PDB ID:3POW), *Mus musculus* calreticulin(MmCRT) (PDB ID: 3RG0), and EhCRT (PDB ID:5HCA), is coordinated by the side chains of five residues including T30, D32, D66, M67, and D332 (**Figure 1B**), which reflects the calcium-binding capacity of TsCRT.

The PDB coordinates of TsCRT^△^ were uploaded to the DALI server (29, 40) for structural alignment against the two parasitic protozoan CRT structures solved so far, namely, EhCRT (PDB ID: 5HCA) and TcCRT(PDB ID: 5HCF), with r.m.s.d. values being 1.5 Å and 2.1 Å, respectively (41). Alignment of TsCRT^△^ with the two existing protozoan CRT structures revealed that six regions showed structural differences, namely, Helix1 (H1), Sheet2 (S2), Helix3 (H3), Sheet4 (S4), Sheet5 (S5), and Helix6 (H6) (**Figures 1C, D**).

The region H1 (Residues 35–40: DAWKER), close to the N terminus of TsCRT^△^, forms a short α helix in TsCRT^△^ while the α helix is replaced by a more flexible loop on the corresponding location of TcCRT and EhCRT. As for the immediately following region S2 (Residues 42–43: VQ), TsCRT^△^ remains a loop instead of a rigid motif while TcCRT and EhCRT form a β sheet. The region H3 (Residues 122–125: DLED) of TsCRT^△^ is shaped as a flexible loop in contrast to the counterpart of TcCRT and EhCRT, which form a more rigid α helix (**Figure 1C**). The region S4 (Residues 133–143: RIMFGPDICGP) forms a long β sheet as TcCRT, while the EhCRT is shaped as a large loop. The H6 region (Residues 349–354: KQLEAL) represents the long α helix at the C terminus. Although the long α helix is straight in all protozoan CRTs, it bends abruptly at Q350 in TsCRT^△^. Immediately preceding the C-terminal α helix are two small β sheets at the S5 site (Residues 332–334: DNI) of TsCRT^△^, taking the place of a larger β sheet at the corresponding S5 site of the two protist structures (**Figure 1D**). Sequence alignments of the six regions all demonstrate conservation to various degrees, except for H6. The absence of conservation in region H6 may reflect that the long C-terminal α helix of CRTs may be equipped with flexibility to perform different functions.

### TsCRT^Δ^ inhibits classical complement activation through binding to C1q

**Figure 2:**
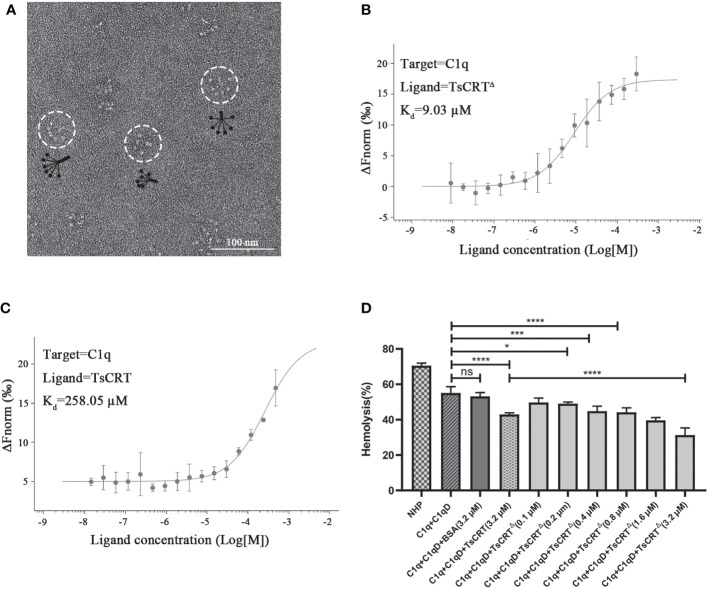
TsCRT^Δ^ inhibits the initiation of classical complement pathway through binding to C1q. **(A)** Well-folded C1q was confirmed by negative staining electron microscopy. **(B, C)** The binding affinity between TsCRT^Δ^/TsCRT and C1q revealed by *K*_d_ values were measured with MST. **(D)** TsCRT^Δ^ inhibited C1q-initiated sheep red blood cell hemolysis in a dose-dependent manner (0.1 µM, 0.2 µM, 0.4 µM, 0.8 µM, 1.6 µM, or 3.2 µM). NHP, normal human plasma; C1q-D, C1q-deficient serum; BSA, bovine serum albumin. Each experiment was repeated three times. Data are shown as the mean ± SDs (ns, no significance, *p < 0.05, ***p < 0.001, ****p < 0.0001).

To determine whether TsCRT^△^ possessed the same complement modulation function as the full-length TsCRT, biochemical and hemolysis assays were conducted. The C1q used in this functional assay was confirmed to possess the correct conformation and structure through negative staining electron microscopy (**Figure 2A**). Overall, C1q is composed of six heterotrimers resembling a tulip flower, each consisting of an N-terminal collagen-like fragment and a C-terminal globular region (42). The binding assay with C1q revealed that the binding affinity of TsCRT^△^ to C1q was even stronger than that of the parental TsCRT, based on the *K*_d_ values measured using MST assay. The *K*_d_ value of TsCRT^△^ binding to C1q was calculated as 9.03 µM, which was significantly lower than that of full-length TsCRT (258.05 µM, **Figures 2B, C**). In addition, TsCRT^△^ inhibited red blood cell hemolysis when pre-incubated with C1q in a dose-dependent manner (**Figure 2D**), indicating that TsCRT^△^ inhibits the initiation of complement activation through binding to C1q and interferes with the formation of membrane attack complex.

### TsCRT^△^ competes with IgG to bind to C1q

To determine whether the TsCRT-binding site on C1q overlap with its IgG/IgM-binding site, the hydrogen/deuterium exchange (HDX-MS) assay and molecular docking were performed, with the former assay to locate specific peptides participating in the interaction and the latter to analyze the specific residues engaged in the binding activity (37, 43).

**Figure 3:**
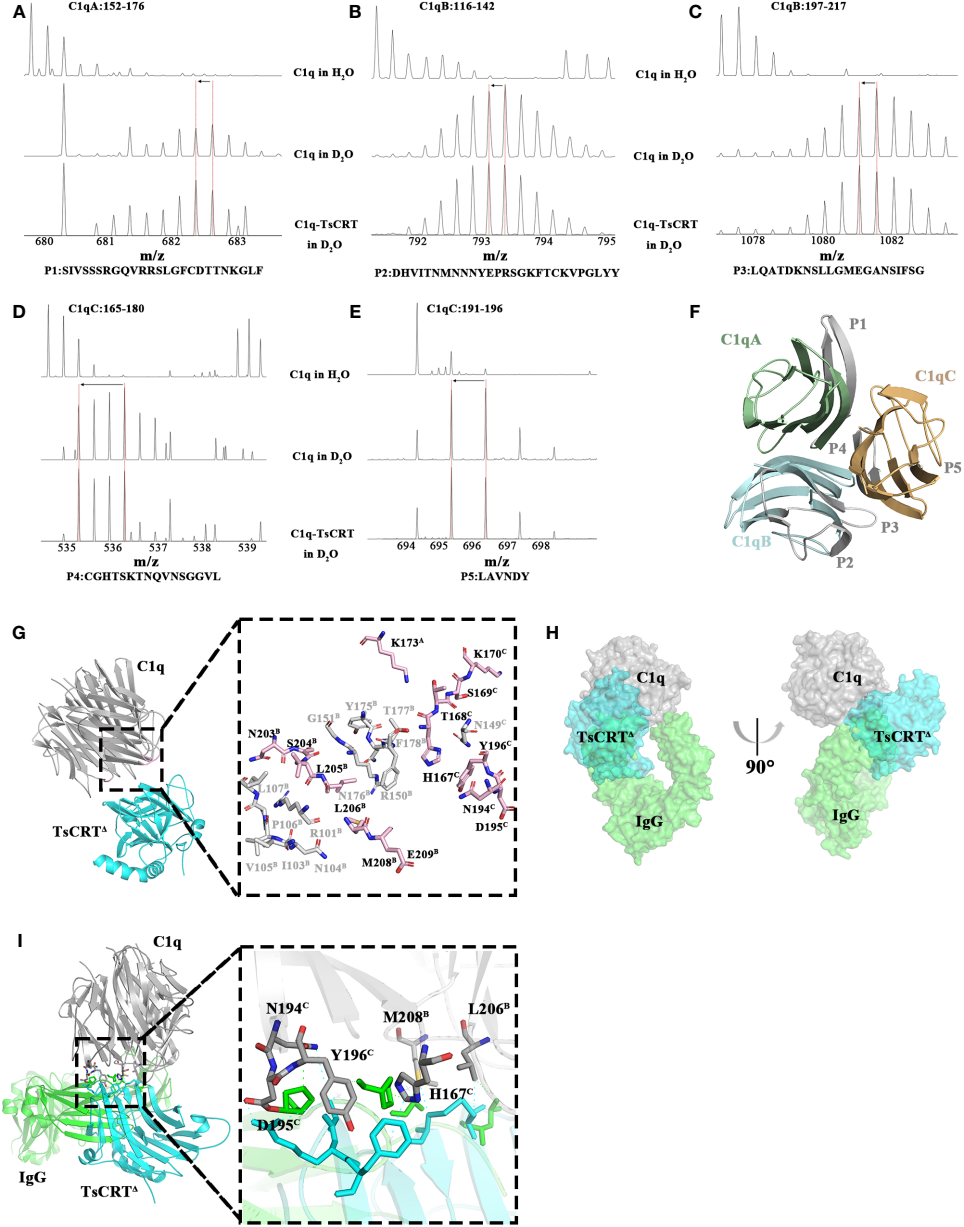
Regions of C1q involved in the interaction with TsCRT^Δ^. **(A–E)** The results of the H/D exchange experiments localized peptides of C1q that are potentially engaged in binding activity to TsCRT^Δ^. The first row represents the unexchanged pattern for peptides of individual C1q in H_2_O, the second row represents the shift pattern for peptides of individual C1q in D_2_O, and the last row represents the shift pattern for peptides of C1q incubated with TsCRT^Δ^ in D_2_O. The *x*-axes represent the mass-to-charge ratio of peptides. The red dashed vertical lines indicate the mass-to-charge ratio for each peptide in different samples. Shift patterns for peptide P1 **(A)**, P2 **(B)**, P3 **(C)**, P4 **(D)**, and P5 **(E)**. **(F)** Cartoon representation of C1q. Regions of C1q in gray are involved in TsCRT^Δ^ interaction based on the H/D exchange results. C1qA in pale green, C1qB in pale cyan, and C1qC in light orange. **(G)** The molecular docking model of TsCRT^Δ^ and C1q-GR is acquired using Alphafold2 (left) and the residues on C1q involved in interaction cross-referenced between molecular docking and H/D exchange experiments (right). C1q in gray, TsCRT^Δ^ in cyan. The residues involved in TsCRT^Δ^ interaction on C1q by molecular docking displayed as sticks, where light pink or gray sticks indicate agreement or disagreement with H/D exchange experiments, respectively(right). **(H)** Three-dimensional superposition between TsCRT^Δ^-C1q and IgG-C1q (PDB ID: 6FCZ) is shown on the surface; TsCRT^Δ^ in cyan, IgG in green, and C1q in gray. The overlapping green and blue regions represent regions of conflict on TsCRT^Δ^/IgG binding to C1q. **(I)** Overall tertiary structure of TsCRT^Δ^ and IgG interacting with C1q (left) and the magnified details of the overlapping interaction interface between TsCRT^Δ^-C1q and IgG-C1q (right). Six residues of C1q shown in gray sticks may participate in the interaction with both TsCRT^Δ^ and IgG. Residues of IgG involved in the interaction with C1q indicated in green dashed line were shown in green sticks; Residues of TsCRT^Δ^ involved in the interaction with C1q indicated in cyan dashed line were shown in cyan sticks.

To achieve this, C1q was incubated with TsCRT^△^ to build an artificial complex, then digested with pepsin for HDX-MS assay targeting C1q. The deuterium exchange value of the five peptides derived from C1q, including P1 (Residues 152^A^–176^A^: SIVSSSRGQVRRSLGFCDTTNKGLF), P2 (Residues 116^B^–142^B^: DHVITNMNNYEPRSGKFTCKVPGLYY), P3 (Residues 197^B^–217^B^: LQATDKNSLLGMEGANSIFSG), P4(residues 165^C^–180^C^: CGHTSKTNQVNSGGVL), and P5 (Residues 191^C^–196^C^: LAVNDY) demonstrated a decreasing trend when incubated with TsCRT^△^. As demonstrated in **Figures 3A–E**, the peak axis location (where the red dashed vertical lines indicate), representing the mass-to-charge ratio of each peptide from C1q shift to the left from a larger one of C1q in D_2_O to a smaller one of C1q incubated with TsCRT^△^ in D_2_O (indicated by left arrows), representing fewer H/D exchanges in the peptides occur. This suggests that the structural environment around the five peptides changed, which is highly possibly attributable to their engagement in binding activity. Notably, all the five peptides are located on the GR of C1q (namely C1q-GR) (shown in gray in **Figure 3F**). To further elaborate whether these five peptides are involved in the interaction with TsCRT, we performed virtual molecular docking of TsCRT^△^ with C1q-GR using Alphafold2. The chosen docking model of the highest score predicted that 27 residues on C1q (K173^A^, R101^B^, I103^B^, N104^B^, V105^B^, P106^B^, L107^B^, R150^B^, G151^B^, Y175^B^, N176^B^, T177^B^, F178^B^, N203^B^, S204^B^, L205^B^, L206^B^, M208^B^, E209^B^, N149^C^, H167^C^, T168^C^, S169^C^, K170^C^, N194^C^, D195^C^, and Y196^C^) were involved in the interaction analyzed by Chimera (shown in gray and pink in **Figure 3G**). Most of them fall on chain B and chain C of C1q-GR. Aligned with the five peptides of C1q—as identified through the HDX-MS assay—with the highest probability of being involved in binding to TsCRT, total 14 residues (K173^A^, N203^B^, S204^B^, L205^B^, L206^B^, M208^B^, E209^B^, H167^C^, T168^C^, S169^C^, K170^C^, N194^C^, D195^C^, and Y196^C^) are overlapped in both HDX-MS assay and the docking prediction. These 14 overlapped residues are included in the four peptides, P1, P3, P4, and P5 except for P2 (shown in pink in **Figure 3G**). In other words, at least one residue of P1, P3, P4, and P5 is engaged in the interaction.

The complex structure of Fc fragment of IgG and C1q-GR (IgG-C1q) has been resolved based on a 7 Å EM map (PDB ID: 6FCZ) which provides a clear visualization of the binding of the Fc region of IgG to C1q, with chain B and chain C of C1q-GR as the major players (44). These findings are consistent with our HDX-MS results, which show that four of the five potential binding peptides of C1q are located on chain B and chain C of C1q-GR. To obtain a clear picture of the overall interaction activity, the two binding complexes including both TsCRT^△^-C1q and IgG-C1q were superimposed; this demonstrated that TsCRT^△^ (cyan) clashes with IgG (green) in binding to C1q (gray) (**Figure 3H**), indicating that TsCRT^△^ binding to C1q sterically interferes with its binding activity to IgG-Fc and further hinder the initiation of the classical complement system.

Further analysis of the particular IgG-binding sites on C1q showed that 15 residues on chain B (T102^B^, N104^B^, V105^B^, P106^B^, R114^B^, D116^B^, H117^B^, P128^B^, R129^B^, R150^B^, P178^B^, L206^B^, M208^B^, G210^B^, and A211^B^) and seven residues on chain C (N107^C^, C151^C^, C165^C^, H167^C^, N194^C^, D195^C^, and Y196^C^) of C1q-GR were predicted to be involved in the interaction with IgG (**Supplementary Figure S2**). Cross-alignment with the 14 residues of C1q that participate in the TsCRT^△^-C1q interaction revealed six residues overlapped, namely, L206^B^, M208^B^, H167^C^, N194^C^, D195^C^, and Y196^C^ (**Figure 3I**). This finding indicates that these six residues are engaged in binding activity with both IgG and TsCRT^△^. The results show TsCRT^△^ and IgG can competitively bind to C1q, which may interpret the inhibition of TsCRT^△^ on the activation of antigen-antibody complex-induced C1q-based classical complement activation.

### Identification of C1q-binding sites on TsCRT

**Figure 4:**
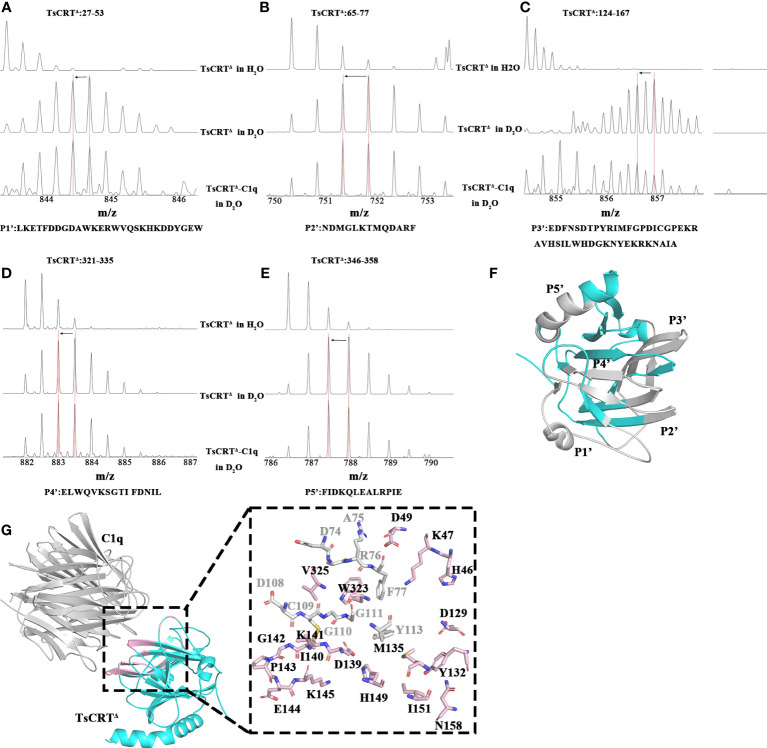
Regions of TsCRT^Δ^ involved in interaction with C1q. **(A–E)** The regions on TsCRT^Δ^ that are involved in the C1q interaction were identified by H/D exchange mass spectrometry. There are three shift patterns for each peptide, where the first row represents the unexchanged pattern of peptides from individual TsCRT^Δ^ in H_2_O, the second row represents the shift pattern of peptides from individual TsCRT^Δ^ in D_2_O, and the last row represents shift patterns of peptides from TsCRT^Δ^ incubated with C1q in D_2_O. The *x*-axes represent the mass-to-charge ratio of peptides. The red dashed vertical lines indicate the mass-to-charge ratio for each peptide in different samples. When the mass-to-charge ratio of peptides from TsCRT^Δ^ incubated with C1q shifts to the left compared with that of peptides from individual TsCRT^Δ^, less H/D exchanges occur in the peptides and vice versa. Shift patterns for peptide P1′ **(A)**, P2′ **(B)**, P3′ **(C)**, P4′ **(D)**, and P5′ **(E)**. **(F)** Regions in gray represent peptides of TsCRT^Δ^ with decreasing hydrogen/deuterium exchanging rates. TsCRT^Δ^ in cyan. **(G)** The molecular docking model of TsCRT^Δ^ and C1q-GR is acquired using Alphafold2 (left) and the residues on TsCRT^Δ^ involved in interaction cross-referenced between molecular docking and H/D exchange experiments (right). Regions of TsCRT^Δ^ involved in binding to C1q by molecular docking are shown in cartoon form in light pink (left). The 27 residues of TsCRT^Δ^ involved in binding to C1q analyzed by molecular docking are shown in sticks, where light pink sticks indicate consistency with H/D exchange experiments and gray sticks indicate inconsistency (right).

Although plenty of scientific endeavors have been made in elucidating the involvement of calreticulin in immune evasion mechanisms of parasites within the host, the specific sites on TsCRT that are engaged in binding to C1q remain unclear. To identify these sites, TsCRT^△^ was incubated with C1q to build an artificial complex for H/D exchange assay targeting TsCRT^△^. After digestion with pepsin, five peptides of TsCRT^△^, namely, P1′(Residues 27–53: LKETFDDGDAWKERWVQSKHKDDYGEW), P2′(Residues 65–77: NDMGLKTMQDARF), P3′(Residues 124–167: EDFNSDTPYRIMFGPDICGPEKRAVHSILWHDGKNYEKRKNAIA), P4′(Residues 321–335: ELWQVK- SGTIFDNIL), and P5′ (Residues 346–358: FIDKQLEALRPIE) demonstrated the shift of the mass-to-charge ratio to the left (indicated by the left arrow), suggesting that the structural environment around the complex has changed because of the direct molecule-molecule interaction on the interface or the surrounding structural change (away from the interface) (**Figures 4A–E**). The corresponding location of the five peptides in the TsCRT^△^ structure was demonstrated (**Figure 4F**).

**Figure 5:**
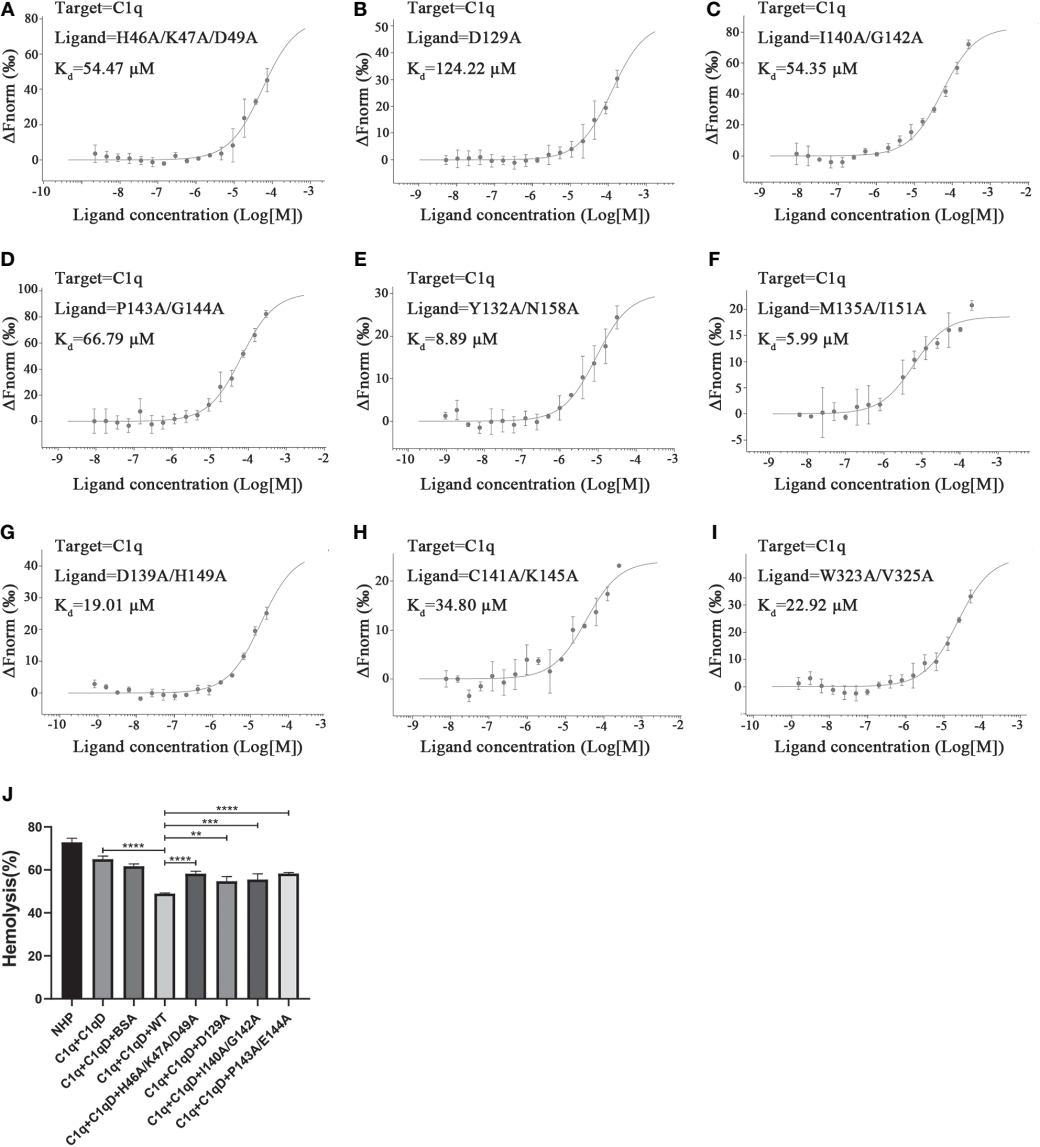
Determination of key residues within TsCRT^Δ^ involved in its binding to C1q. **(A–I)** The dissociation constant (*K*_d_) representing the affinity between nine mutants of TsCRT^Δ^ and C1q measured using microscale thermophoresis (MST) assays. The *K*_d_ value was significantly increased in the first four mutants **(A–D)**, indicating these mutated residues including H46, K47, D49, D129, I140, G142, P143, and G144 play important roles in the interaction of TsCRT^Δ^ with C1q. **(J)** When C1q was incubated with four mutants of TsCRT^Δ^ (3.2 µM), the inhibition of hemolysis of sheep erythrocytes was significantly reduced compared to the prototype TsCRT^Δ^. NHP, normal human plasma; C1q-D, C1q-deficient serum; BSA, bovine serum albumin. Each experiment was repeated three times. Data are shown as the mean ± SDs (**p < 0.01, ***p < 0.001, ****p < 0.0001).

Analysis of the interface of the TsCRT^△^-C1q docking model using Chimera indicates that 27 residues in TsCRT^△^ are engaged in the interaction (shown in gray and pink in **Figure 4G**: H46, K47, D49, D74, A75, R76, F77, D108, C109, G110, G111, Y113, D129, Y132, M135, D139, I140, C141, G142, P143, E144, K145, H149, I151, N158, W323, and V325). Of these, 18 residues are included in the five peptides of TsCRT engaged in interaction with C1q, as determined by HDX-MS assay (shown in pink in **Figure 4G**). To further confirm whether these residues are involved in the binding of TsCRT^△^ to C1q, these 18 residues were mutated in the sequence of TsCRT^Δ^ individually or in combination to generate nine mutants as below (located in the corresponding peptide): H46A/K47A/D49A(P1′), D129A(P3′), M135A/I151A(P3′), Y132A/N158A(P3′), D139A/H149A(P3′), I140A/G142A(P3′), C141A/K145A(P3′), P143A/E144A(P3′), and W323A/V325A(P4′) according to their conformational proximity. These nine TsCRT^Δ^ mutants were used to perform MST to identify changes in their binding affinity to individual C1q compared with the original TsCRT^Δ^ (prototype). The binding affinity to C1q for four of the nine mutants namely H46A/K47A/D49A(P1′), D129A(P3′), I140A/G142A(P3′) and P143A/E144A(P3′), was reduced up to five folds in terms of the *K*_d_ value (54.47 µM, 124.22 µM, 54.35 µM, and 66.79 µM, respectively, compared with the *K*_d_ value of 9.03 µM (**Figure 2B**) for the prototype (**Figures 5A–D**). It is evident that residue D129 plays most substantial role in the interaction. This is because it undergoes the largest reduction in binding affinity compared with the other mutants and the prototype (**Figures 5E–I**). The C1q-involved hemolysis assay also showed that all four mutants demonstrated significantly reduced inhibition relative to the prototype (**Figure 5J**), functionally confirming that these residues are involved in C1q binding and the subsequent inhibition of C1q-involved classical complement activation.

### C1q-binding peptide from TsCRT inhibits C1q-initiated complement activation

**Figure 6:**
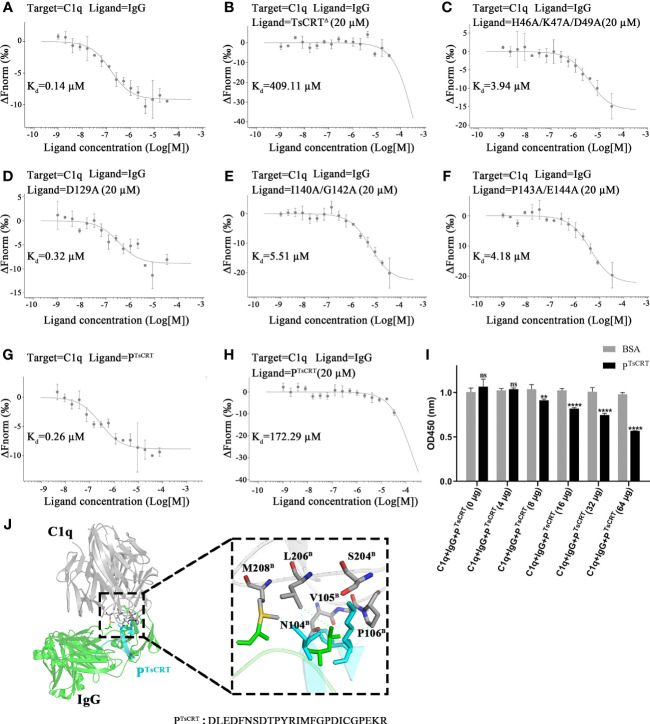
Identification of residues of TsCRT involved in the competitive binding of C1q with IgG. **(A–F)** TsCRT^Δ^ inhibits the interaction between C1q and IgG **(A, B)** measured by quantitatively competitive binding MST assays. The inhibition of the interaction, however, was significantly attenuated when the TsCRT^Δ^ mutants containing eight residue mutations, namely, H46, K47, D49, D129, I140, G142, P143, and E144, replaced the prototype TsCRT^Δ^ **(C–F)**. In the thermophoresis assays, IgG was incubated with fluorescently labeled C1q alone **(A)** or with fluorescently labeled C1q and 20 µM TsCRT^Δ^ **(B)** or different mutants **(C–F)**. Dissociation curves fit to the data to calculate the *K*_d_ values are shown. **(G, H)** P^TsCRT^ inhibits the interaction between C1q and IgG as confirmed by qualitatively competitive binding MST assays. In the thermophoresis assays, P^TsCRT^ was incubated with C1q alone to obtain the *K*_d_ value for binding affinity **(G)**. P^TsCRT^(20 µM) was added to C1q incubated with IgG for competitive inhibition ability **(H)**. **(I)** P^TsCRT^ inhibits the interaction between C1q and IgG as confirmed by qualitatively competitive binding ELISA. BSA was used as a control. BSA, bovine serum albumin. Each experiment was repeated three times. Data are shown as the mean ± SDs (ns, no significance, **p < 0.01, ****p < 0.0001). **(J)** Overall tertiary structure of P^TsCRT^ and IgG interacting with C1q (left) and the magnified details of the interaction interface between P^TsCRT^ and C1q in the presence of IgG (right). P^TsCRT^ mainly interacts with C1q through six residues displayed as sticks, namely, N104^B^, V105 ^B^, P106 ^B^, S204 ^B^, L206^B^. and M208^B^. Residues of IgG involved in the interaction with C1q indicated in green dashed line were shown in green sticks; Residues of P^TsCRT^ involved in the interaction with C1q indicated in cyan dashed line were shown in cyan sticks.C1q in gray, IgG in green, and P^TsCRT^ in cyan.

The above results show that four TsCRT^Δ^ mutants (H46A/K47A/D49A, D129A, I140A/G142A, and P143A/E144A) exhibit a significant reduction in C1q-binding affinity, indicating that they are key residues involved in binding to C1q. To further determine whether these mutants show reduced competitive binding to IgG, these mutants, as well as TsCRT^△^(gradient-diluted) were added to the mixture of pre-incubated fluorescently labeled C1q and IgG. As expected, TsCRT^△^ significantly inhibited C1q binding to IgG (*K*_d_ = 409.11 µM) up to 4,000 folds compared with the control without TsCRT^△^ (*K*_d_ = 0.14 µM) (**Figures 6A, B**). However, when C1q was pre-incubated with the nine TsCRT^△^ mutants, four of them demonstrated significantly reduced inhibition of binding to IgG (*K*_d_ value being up to 400 folds higher than when incubated with prototype TsCRT^△^), indicating that these eight mutated residues in the four mutants play an important role in inhibiting the binding of C1q to IgG. The results further confirm that the original eight residues within the four mutants play an important role in TsCRT’s binding to C1q and competitively inhibit the binding of IgG to C1q (**Figures 6C–F**). The mutant D129A revealed lowest inhibition of the binding of C1q to IgG (*K*_d_ = 0.32 µM), which is similar to that for the control without TsCRT^△^ (*K*_d_ = 0.14 µM) (**Figures 6B, D**). These findings suggest that D129 is the most important residue involved in the binding of TsCRT to C1q.

To further confirm the key role of these residues in the binding of TsCRT to C1q, a new C1q-binding peptide containing three key residues (I140, P143, and D129) within the N-terminal globular domain of TsCRT based on the mutant results above was constructed as P^TsCRT^ (Residues 122–146: DLEDFNSDTPYRIMFGPDICGPEKR) and synthesized. Significantly, the C1q-binding peptide P^TsCRT^ exhibited even stronger binding affinity to C1q (*K*_d_ = 0.26 µM) than the original TsCRT^△^ did (*K*_d_ = 9.03 µM), as determined by MST assay (**Figure 6G**).

In addition, the P^TsCRT^ peptide exhibited significant inhibition on the binding of C1q to IgG, as determined by MST assay, of up to 1,200 folds (*K*_d_ value increased from 0.14 µM to 172.29 µM) (**Figures 6A, H**). The competitive binding of C1q to IgG was also confirmed by competitive binding assay (ELISA) with IgG or BSA coated on the plate. Incubation with P^TsCRT^ significantly inhibited C1q binding to IgG in a dose-dependent manner (**Figure 6I**).

Based on the structural analysis demonstrated in **Figure 6J**, P^TsCRT^ is located in the binding site of TsCRT to C1q through six residues, namely, N104^B^, V105^B^, P106^B^, S204^B^, L206^B^, and M208^B^ within the B chain of C1q. Interestingly, two of them, L206 and M208, were among the six residues on C1q involved in both binding to TsCRT and IgG, as shown in **Figure 3I**. Therefore, P^TsCRT^ can be reasonably postulated to compete with IgG to bind to C1q. All results indicate that the peptide P^TsCRT^ is the C1q-binding site on TsCRT that not only strongly binds to C1q, but also competitively inhibits the binding of IgG to C1q to abrogate C1q/IgG-initiated classical complement activation.

## Discussion

It has been shown that CRTs are highly conserved in several parasite species, from protozoa (*Trypanosoma*, *Amoeba*, and *Leishmania*) to helminths (*Echinococcus*, *Opisthorchis viverrini*, *Brugia malayi* and *Necator americanus*), and play important roles in the adaptation of parasites to the hostile environments and escape from host immune responses (4, 7, 21, 45, 46). Our previous studies have determined that TsCRT can bind to human complement C1q and inhibit C1q-initiated classical complement activation to enable the parasite to evade host first-line immune attack (15, 16). However, the structure of TsCRT has not been resolved, and the structure-related interaction between TsCRTs and C1q, and its impact on the C1q-initiated complement activation, remains unknown. Because of the inherent flexibility of CRTs, the recombinant protein of the full-length TsCRT failed to form a crystal in the current sequence form. To resolve its crystal structure, it is necessary for the recombinant construct of TsCRT to be redesigned and screened for successful crystallization. TsCRT is composed of a globular domain at both the N and C termini with a flexible arm-like P domain inserted in the middle that may interfere with the crystallization according to predicted model by Alphafold2 (7). Therefore, a truncated TsCRT^Δ^ was constructed by deleting the P-domain and part of the C-terminal globular domain, linked with a flexible GSG linker. The recombinant proteins of TsCRT^Δ^ as well as full-length TsCRT were successfully expressed in *E. coli* and the three-dimensional structure of TsCRT^Δ^ was resolved, as the first helminth calreticulin structure, through x-ray crystallography. Similar to other parasite CRTs, TsCRT^△^ is composed of three α helices,16 β sheets, and several loops between them, except for six regions with differences from protozoan CRTs (TcCRT and EhCRT), namely, H1, S2, H3, S4, S5, and H6 (**Figures 1C, D**). A calcium ion binding niche is observed in TsCRT coordinated by the side chains of five residues, namely, T30, D32, D66, M67, and D332, corresponding to its calcium-binding capacity (**Figure 1B**).

Surprisingly, the truncated TsCRT^Δ^ conferred even better binding affinity to C1q than the full-length TsCRT did (**Figures 2B, C**), possibly because the C1q binding site is mainly located at the globular domain that remains in the TsCRT^Δ^ and the truncated form of TsCRT may has less binding interference from unnecessary parts of the protein. The functional assay with antibody-sensitized sheep blood cells also revealed that, like the parental TsCRT, TsCRT^Δ^ retained its inhibitory effect on C1q-initiated classical complement activation in a dose-dependent manner (**Figure 2D**).

To activate the classical pathway, it is necessary for C1q to interact with the antigen-antibody(IgG/IgM) complex to expose the C1r and C1s interacting sites. We hypothesize that TsCRT binds to C1q to compete with its binding site to IgG/IgM. As a result, the interaction between TsCRT with C1q may create steric hindrance to its binding to IgG/IgM. To determine whether TsCRT^Δ^ binds to C1q where is involved in the binding to IgG-Fc, the TsCRT^Δ^-binding site (five peptides including P1–P5) on C1q was determined by HDX-MS and the potential residues on C1q that are involved in the binding to TsCRT^Δ^ were predicted by molecular docking using Alphafold2. Combining the two methods, 14 residues on C1q (K173^A^, N203^B^, S204^B^, L205^B^, L206^B^, M208^B^, E209^B^, H167^C^, T168^C^, S169^C^, K170^C^, N194^C^, D195^C^, and Y196^C^) are predicted to be involved in its binding to TsCRT^Δ^ (**Figure 3**). To determine the IgG-binding sites on C1q, the real IgG-C1q complex structure (PDB ID: 6FCZ) was used for analysis and it was found that 22 residues in C1q (15 residues on chain B and seven residues on chain C of C1q-GR) are involved in the interaction with IgG (44). Cross-alignment with the 14 residues of C1q that participate in the TsCRT^△^-C1q interaction suggested that six residues on C1q, namely, L206^B^, M208^B^, H167^C^, N194^C^, D195^C^, and Y196^C^, are involved in the binding activity with both IgG and TsCRT^△^ (**Figure 3I**). The results suggest that TsCRT^△^ and IgG can bind to the same sites on C1q, which may explain the competitive binding of TsCRT on C1q with IgG to inhibit antigen-antibody complex-induced C1q-initiated classical complement activation. The resolved TsCRT^Δ^ structure was used to determine the C1q-binding sites on TsCRT by using different biochemical and functional assays. HDX-MS assay demonstrated that five peptides located at the N terminus (P1′27–53, P2′65–77, and P3′124–167) and the C terminus (P4′321–335 and P5′346–358) of TsCRT^△^ were involved in binding to C1q, because the mass-to-charge ratio reduced in the D_2_O condition due to the molecule–molecule interaction on the interface (**Figures 4A–E**). As shown in **Figures 1D** and **E**, there are six regions, namely, H1, S2, H3, S4, S5, and H6, where the sequence and structure of TsCRT^△^ differs from two protozoan calreticulin, TcCRT, and EhCRT. The difference in sequence or structure may reflect the different functions of helminthic CRTs and protozoan CRTs in terms of interaction with hosts. Coincidentally, four of five peptides of TsCRT^△^ identified by the HDX-MS assay fall into these regions (as H1 and S2 contained in P1′, H3 and S4 contained in P3′, and S5 and H6 contained in P4′ and P5′), which may account for the different binding modes between helminth CRTs and protozoan CRTs. Therefore, the interplay between TcCRT/EhCRT and C1q may be completely or at least partially different from that involving TsCRT.

Through Chimera analysis of the docking model of TsCRT^△^-C1q, 27 residues in TsCRT^△^ were predicted to be involved in the interaction with C1q-GR. Interestingly, 18 of them are included in the five peptides of TsCRT^△^ that interact with C1q, as determined by the HDX-MS assay (**Figure 4G**). To further pin down the exact residues involved in the binding of TsCRT^△^ to C1q, nine mutants of TsCRT^Δ^ were generated, containing different mutations on these 18 residues. MST assay was performed on these nine mutants to detect changes in their binding affinity to C1q compared with the prototype; four of them demonstrated significantly reduced binding affinity to C1q compared with the prototype TsCRT^Δ^ (**Figures 5A–I**), with D129A showing the largest reduction, indicating that D129 is the key residue involved in binding to C1q (**Figure 5C**). The important residues mutated in these four mutants all are located at the N-terminal globular domain (H46, K47, D49, D129, I140, G142A, P143, and E144). Functional assay with these four mutants also revealed a reduction in C1q-involved hemolysis compared with that for the prototype TsCRT^△^ (**Figure 5J**), confirming that these residues, especially D129, are involved in C1q binding and subsequent inhibition of C1q-involved classical complement activation.

To further determine whether these key residues within these mutants competitively change the binding ability of C1q to IgG, a competitive MST assay with fluorescently labeled C1q was performed in the presence of both TsCRT^△^ mutants and IgG. As expected, TsCRT^△^ significantly inhibited C1q binding to IgG. However, the same four mutants, especially D129A, significantly compromised their inhibition in C1q’s binding to IgG (**Figure 6**), further confirming that these eight residues within the four mutants are involved in the binding to C1q as well as in competitively inhibiting the binding of IgG to C1q. This functionally explains the involvement of TsCRT in the inhibition of C1q-initiated complement attack, and the eight residues within TsCRT, especially D129, play an important role in this function.

Sequence alignment between TsCRT and other four helminth-produced CRTs, namely, *H. contortus* calreticulin (HcCRT), *N. americanus* calreticulin (NaCRT), *B. malayi* calreticulin (BmCRT), and *O. viverrini* calreticulin (OvCRT)—which also participate in complement evasion through interacting with C1q (21, 47, 48)—revealed that seven of eight key residues are conserved in all helminth CRTs (**Supplementary Figure S3**). Coincidently, the Yadav group analyzed a docking model of BmCRT binding to C1q, using a predicted structure model, from which 37 residues were considered to be involved in the interaction with C1q; 20 of them being on the N-domain, including the eight conserved key residues identified on TsCRT^△^ (20). This supports the assumption that helminth CRTs are highly conserved in terms of structure and the structural basis of complement evasion. This finding needs further confirmation with more calreticulin structures of different species, especially helminths, being determined.

Based on the key role of these residues in the binding of TsCRT to C1q and consequent inhibition of its binding to IgG, a 24 amino acid-peptide containing three key residues (D129, I140, and P143) within the N-terminal globular domain of TsCRT was constructed as the C1q-binding peptide P^TsCRT^. Surprisingly the synthesized C1q-binding peptide P^TsCRT^ revealed even stronger binding affinity to C1q (*K*_d_ = 0.26 µM) (**Figure 2A**) than the prototype TsCRT^△^ did (*K*_d_ = 9.03 µM) (**Figure 6G**); it also significantly inhibited IgG binding to C1q, as shown by both competitive MST and ELISA assays (**Figures 6H, I**).

Based on molecular docking and structural analysis, P^TsCRT^ was found to be located in the binding site of TsCRT to C1q through six residues N104^B^, V105^B^, P106^B^, S204^B^, L206^B^, and M208^B^ on C1q (**Figure 6J**). Interestingly, L206^B^ and M208^B^ are among the six residues on C1q involved in binding to both TsCRT and IgG, as shown in **Figure 3I**. As a result, P^TsCRT^ is structurally justified to be able to compete with IgG to bind to C1q and inhibit antigen-antibody/C1q-initiated classical complement activation. Potentially, this C1q-binding peptide can be further developed as a vaccine or drug target for preventing or treating trichinellosis that threats millions of people living in the endemic region. In addition, with the inhibition effect on initiation of complement activation, P^TsCRT^ can be taken as a complement modulator to treat autoimmune diseases caused by complement hypersensitivity (49).

In this study, it is the first to solve the structure of helminth *T. spiralis*-produced calreticulin (TsCRT) by removing the flexible arm-like P domain, which remains even stronger C1q binding activity than full-length TsCRT. Accordingly, the fact that TsCRT shares the same binding region on C1q with IgG was identified, structurally elucidating the molecular mechanism underlying TsCRT-involved complement evasion during the establishment of *T. spiralis* parasitism in hosts. Finally, a 24 amino acid-peptide called P^TsCRT^ was constructed that displayed strong C1q-binding activity and inhibition of C1q-IgG–initiated classical complement activation. This study demonstrates, for the first time, the structurally related function of helminth-derived calreticulin involved in the immune evasion, providing an approach to develop helminth-derived bifunctional molecules to prevent or treat infection of parasitic diseases or autoimmune diseases related to the complement hypersensitivity.

## Data availability statement

The HDX-MS datasets presented in this study can be found in online figshare repositories (DOI: https://doi.org/10.6084/m9.figshare.25515391.v1). The coordinates and structure factors have been deposited and released in the Protein Data Bank under ID code 8XVF.

## Ethics statement

The studies involving humans were approved by the Institutional Review Board (IRB) of Capital Medical University. The studies were conducted in accordance with the local legislation and institutional requirements. The participants provided their written informed consent to participate in this study.

## Author contributions

ZJ: Conceptualization, Funding acquisition, Investigation, Software, Supervision, Writing – original draft, Writing – review & editing. WY: Data curation, Methodology, Software, Writing – original draft. JL: Data curation, Formal Analysis, Methodology, Writing – original draft. MZ: Data curation, Methodology, Writing – original draft. BZ: Writing – review & editing. LY: Data curation, Software, Writing – original draft. ZM: Data curation, Methodology, Software, Writing – original draft. YC: Writing – review & editing. XT: Methodology, Writing – original draft. SS: Methodology, Writing – original draft. JH: Methodology, Writing – original draft. XZ: Funding acquisition, Supervision, Writing – review & editing.

## References

[1] Holers VM. Complement and its receptors: new insights into human disease. Annu Rev Immunol. (2014) 32:433–59. doi: 10.1146/annurev-immunol-032713-120154 24499275

[2] Kemper C Ferreira VP Paz JT Holers VM Lionakis MS Alexander JJ. Complement: the road less traveled. J Immunol. (2023) 210:119–25. doi: 10.4049/jimmunol.2200540 PMC10038130 36596217

[3] Shao S Sun X Chen Yi Zhan B Zhu X. Complement evasion: an effective strategy that parasites utilize to survive in the host. Front Microbiol. (2019) 10:532. doi: 10.3389/fmicb.2019.00532 30949145 PMC6435963

[4] Esperante D Flisser A Mendlovic F. The many faces of parasite calreticulin. Front Immunol. (2023) 14:1101390. doi: 10.3389/fimmu.2023.1101390 36993959 PMC10040973

[5] Schcolnik-Cabrera A Oldak B Juarez M Cruz-Rivera M Flisser A Mendlovic F. Calreticulin in phagocytosis and cancer: opposite roles in immune response outcomes. Apoptosis. (2019) 24:245–55. doi: 10.1007/s10495-019-01532-0 30929105

[6] Venkatesan A Satin LS Raghavan M. Roles of calreticulin in protein folding, immunity, calcium signaling and cell transformation. Prog Mol subcellular Biol. (2021) 59:145–62. doi: 10.1007/978-3-030-67696-4_7 PMC8969281 34050865

[7] Ramirez-Toloza G Aguilar-Guzman L Valck C Ferreira VP Ferreira A. The interactions of parasite calreticulin with initial complement components: consequences in immunity and virulence. Front Immunol. (2020) 11:1561. doi: 10.3389/fimmu.2020.01561 32793217 PMC7391170

[8] Ramirez-Toloza G Sosoniuk-Roche E Valck C Aguilar-Guzman L Ferreira VP Ferreira A. *Trypanosoma cruzi* calreticulin: immune evasion, infectivity, and tumorigenesis. Trends Parasitol. (2020) 36:368–81. doi: 10.1016/j.pt.2020.01.007 32191851

[9] Ramirez-Toloza G Aguilar-Guzman L Valck C Menon SS Ferreira VP Ferreira A. Is it possible to intervene in the capacity of trypanosoma cruzi to elicit and evade the complement system? Front Immunol. (2021) 12:789145. doi: 10.3389/fimmu.2021.789145 34975884 PMC8716602

[10] Lidani KCF Bavia L Ambrosio AR de Messias-Reason IJ. The complement system: A prey of *trypanosoma cruzi* . Front Microbiol. (2017) 8:607. doi: 10.3389/fmicb.2017.00607 28473804 PMC5397499

[11] Ramirez-Toloza G Ferreira A. *Trypanosoma cruzi* Evades the Complement System as an Efficient Strategy to Survive in the Mammalian Host: The Specific Roles of Host/Parasite Molecules and *Trypanosoma cruzi* Calreticulin. Front Microbiol. (2017) 8:1667. doi: 10.3389/fmicb.2017.01667 28919885 PMC5585158

[12] Vaithilingam A Teixeira JE Miller PJ Heron BT Huston CD. *Entamoeba histolytica* cell surface calreticulin binds human C1q and functions in amebic phagocytosis of host cells. Infection Immun. (2012) 80:2008–18. doi: 10.1128/iai.06287-11 PMC3370593 22473608

[13] Ximenez C Gonzalez E Nieves ME Silva-Olivares A Shibayama M Galindo-Gomez S. *Entamoeba histolytica* and E. dispar Calreticulin: Inhibition of Classical Complement Pathway and Differences in the Level of Expression in Amoebic Liver Abscess. BioMed Res Int. (2014) 2014:127453. doi: 10.1155/2014/127453 24860808 PMC4016843

[14] Yanagawa Y Singh U. Diversity and plasticity of virulent characteristics of *entamoeba histolytica* . Trop Med Infect Dis. (2023) 8(5):255. doi: 10.3390/tropicalmed8050255 37235303 PMC10222173

[15] Zhao L Shao S Chen Yi Sun X Sun R Huang J. *Trichinella spiralis* calreticulin binds human complement C1q as an immune evasion strategy. Front Immunol. (2017) 8:636. doi: 10.3389/fimmu.2017.00636 28620388 PMC5449505

[16] Shao S Hao C Bin Z Zhuang Q Zhao L Chen Yi. *Trichinella spiralis* calreticulin S-domain binds to human complement C1q to interfere with C1q-mediated immune functions. Front Immunol. (2020) 11:572326. doi: 10.3389/fimmu.2020.572326 33329535 PMC7710684

[17] Kasper G Brown A Eberl M Vallar L Kieffer N Berry C. A calreticulin-like molecule from the human hookworm *Necator americanus* interacts with C1q and the cytoplasmic signalling domains of some integrins. Parasite Immunol. (2001) 23:141–52. doi: 10.1046/j.1365-3024.2001.00366.x 11240905

[18] Suchitra S Joshi P. Characterization of *Haemonchus contortus* calreticulin suggests its role in feeding and immune evasion by the parasite. Biochim Et Biophys Acta-General Subj. (2005) 1722:293–303. doi: 10.1016/j.bbagen.2004.12.020 15716049

[19] Naresha S Suryawanshi A Agarwal M Singh BP Joshi P. Mapping the complement C1q binding site in *Haemonchus contortus* calreticulin. Mol Biochem Parasitol. (2009) 166:42–6. doi: 10.1016/j.molbiopara.2009.02.007 19428671

[20] Yadav S Gupta S Selvaraj C Doharey PK Verma A Singh SK. *In silico* and *in vitro* studies on the protein-protein interactions between *brugia malayi* immunomodulatory protein calreticulin and human C1q. PloS One. (2014) 9(9):e106413. doi: 10.1371/journal.pone.0106413 25184227 PMC4153637

[21] Chaibangyang W Geadkaew-Krenc A Smooker PM Tesana S Grams R. Evaluation of *Opisthorchis viverrini* calreticulin for potential host modulation. Acta Tropica. (2018) 187:175–81. doi: 10.1016/j.actatropica.2018.08.009 30098943

[22] Thielens NM Tedesco F Bohlson SS Gaboriaud C Tenner AJ. C1q: A fresh look upon an old molecule. Mol Immunol. (2017) 89:73–83. doi: 10.1016/j.molimm.2017.05.025 28601358 PMC5582005

[23] Schulz K Trendelenburg M. C1q as a target molecule to treat human disease: What do mouse studies teach us? Front Immunol. (2022) 13:958273. doi: 10.3389/fimmu.2022.958273 35990646 PMC9385197

[24] Ferreira V Valck C Sánchez G Gingras A Tzima S Molina MC. The classical activation pathway of the human complement system is specifically inhibited by calreticulin from *Trypanosoma cruzi* . J Immunol. (2004) 172:3042–50. doi: 10.4049/jimmunol.172.5.3042 14978109

[25] Kozlov G Pocanschi CL Rosenauer A Bastos-Aristizabal S Gorelik A Williams DB. Structural basis of carbohydrate recognition by calreticulin. J Biol Chem. (2010) 285:38612–20. doi: 10.1074/jbc.M110.168294 PMC2992293 20880849

[26] Chouquet A Paidassi H Ling WLi Frachet P Houen G Arlaud GJ. X-ray structure of the human calreticulin globular domain reveals a peptide-binding area and suggests a multi-molecular mechanism. PloS One. (2011) 6(3):e17886. doi: 10.1371/journal.pone.0017886 21423620 PMC3057994

[27] Kozlov G Pocanschi CL Rosenauer A Williams DB Gehring K. Structural basis of carbohydrate recognition by calreticulin. Acta Crystallographica a-Foundation Adv. (2011) 67:C548–9. doi: 10.1107/s0108767311086132 PMC2992293 20880849

[28] Pocanschi CL Kozlov G Brockmeier U Brockmeier A Williams DB Gehring K. Structural and functional relationships between the lectin and arm domains of calreticulin. J Biol Chem. (2011) 286:27266–77. doi: 10.1074/jbc.M111.258467 PMC3149320 21652723

[29] Moreau C Cioci G Iannello M Laffly E Chouquet A Ferreira A. Structures of parasite calreticulins provide insights into their flexibility and dual carbohydrate/peptide-binding properties. Iucrj. (2016) 3:408–19. doi: 10.1107/s2052252516012847 PMC5094443 27840680

[30] Fisette O Schroeder GF Schaefer LV. Atomistic structure and dynamics of the human MHC-I peptide-loading complex. Proc Natl Acad Sci United States America. (2020) 117:20597–606. doi: 10.1073/pnas.2004445117 PMC7456110 32788370

[31] Kabsch W. Integration, scaling, space-group assignment and post-refinement. Acta Crystallographica Section D-Biological Crystallogr. (2010) 66:133–44. doi: 10.1107/s0907444909047374 PMC2815666 20124693

[32] McCoy AJ Sammito MD Read RJ. Implications of *AlphaFold*2 for crystallographic phasing by molecular replacement. Acta Crystallographica Section D-Structural Biol. (2022) 78:1–13. doi: 10.1107/s2059798321012122 PMC8725160 34981757

[33] Casanal A Lohkamp B Emsley P. Current developments in Coot for macromolecular model building of Electron Cryo-microscopy and Crystallographic Data. Protein Sci. (2020) 29:1069–78. doi: 10.1002/pro.3791 PMC7096722 31730249

[34] Wienken CJ Baaske P Rothbauer U Braun D Duhr S. Protein-binding assays in biological liquids using microscale thermophoresis. Nat Commun. (2010) 1:100. doi: 10.1038/ncomms1093 20981028

[35] Zhai Z Keereetaweep J Liu H Feil R Lunn JE Shanklin J. Trehalose 6-phosphate positively regulates fatty acid synthesis by stabilizing WRINKLED1. Plant Cell. (2018) 30:2616–27. doi: 10.1105/tpc.18.00521 PMC6241258 30249634

[36] James EI Murphree TA Vorauer C Engen JR Guttman M. Advances in hydrogen/deuterium exchange mass spectrometry and the pursuit of challenging biological systems. Chem Rev. (2022) 122:7562–623. doi: 10.1021/acs.chemrev.1c00279 PMC9053315 34493042

[37] Bryant P Pozzati G Elofsson A. Improved prediction of protein-protein interactions using AlphaFold2. Nat Commun. (2022) 13(1):1265. doi: 10.1038/s41467-022-29480-5 35273146 PMC8913741

[38] Scarff CA Fuller MJG Thompson RF Iadanza MG. Variations on negative stain electron microscopy methods: tools for tackling challenging systems. Jove-Journal Visualized Experiments. (2018) (132):57199. doi: 10.3791/57199 PMC5912373 29443097

[39] Madeira F Pearce M Tivey ARN Basutkar P Lee J Edbali O. Search and sequence analysis tools services from EMBL-EBI in 2022. Nucleic Acids Res. (2022) 50:W276–9. doi: 10.1093/nar/gkac240 PMC9252731 35412617

[40] Holm L. DALI and the persistence of protein shape. Protein Sci. (2020) 29:128–40. doi: 10.1002/pro.3749 PMC6933842 31606894

[41] Holm L Laiho A Törönen P Salgado M. DALI shines a light on remote homologs: One hundred discoveries. Protein Sci. (2023) 32:18. doi: 10.1002/pro.4519 PMC9793968 36419248

[42] Varghese PM Kishore U Rajkumari R. Human C1q regulates influenza A virus infection and inflammatory response via its globular domain. Int J Mol Sci. (2022) 23(6):3045. doi: 10.3390/ijms23063045 35328462 PMC8949502

[43] Tsutsui Y Wintrode PL. Hydrogen/deuterium exchange-mass spectrometry: A powerful tool for probing protein structure, dynamics and interactions. Front Medicinal Chem. (2007) 14(22):2344–58. doi: 10.2174/092986707781745596 17896983

[44] Ugurlar D Howes SC de Kreuk B-J Koning RI de Jong RN Beurskens FJ. Structures of C1-IgG1 provide insights into how danger pattern recognition activates complement. Science. (2018) 359:794–7. doi: 10.1126/science.aao4988 29449492

[45] Sosoniuk-Roche E Cruz P Maldonado I Duaso L Pesce B Michalak M. *In vitro* Treatment of a Murine Mammary Adenocarcinoma Cell Line with Recombinant *Trypanosoma cruzi* Calreticulin Promotes Immunogenicity and Phagocytosis. Mol Immunol. (2020) 124:51–60. doi: 10.1016/j.molimm.2020.05.013 32526557

[46] Xian S Chen L Yan Y Chen J Yu G Shao Y. *Echinococcus multilocularis* calreticulin interferes with C1q-mediated complement activation. Trop Med Infect Dis. (2023) 8(1):47. doi: 10.3390/tropicalmed8010047 36668954 PMC9864966

[47] Logan J Pearson MS Manda SS Choi Y-J Field M Eichenberger RM. Comprehensive analysis of the secreted proteome of adult *Necator americanus* hookworms. PloS Negl Trop Dis. (2020) 14(5):e0008237. doi: 10.1371/journal.pntd.0008237 32453752 PMC7274458

[48] Rahal A Sharma DK Kumar A Sharma N Dayal. D. *In silico* to In vivo development of a polyherbal against *Haemonchus contortus* . Heliyon. (2022) 8(1):e08789. doi: 10.1016/j.heliyon.2022.e08789 35106389 PMC8789534

[49] Trendelenburg M. Autoantibodies against complement component C1q in systemic lupus erythematosus. Clin Trans Immunol. (2021) 10(4):e1279. doi: 10.1002/cti2.1279 PMC8082710 33968409

