# Supplementary material for: Crystal structure of Trichinella spiralis calreticulin and the structural basis of its complement evasion mechanism involving C1q
Source: Front Immunol. 2024 Apr 16;15:1404752. doi: 10.3389/fimmu.2024.1404752 (PMC11059001; doi:10.3389/fimmu.2024.1404752)

**Supporting information captions**

**Supplementary materials**

**Figure S1**

**Figure S2**

**Figure S3**

**Supplementary materials**

**Figure S1** Crystals of TsCRT^Δ^ and the corresponding differaction pattern. (A) The needle-like crystals of TsCRT^Δ^. (B) The corresponding diffraction pattern is displayed.


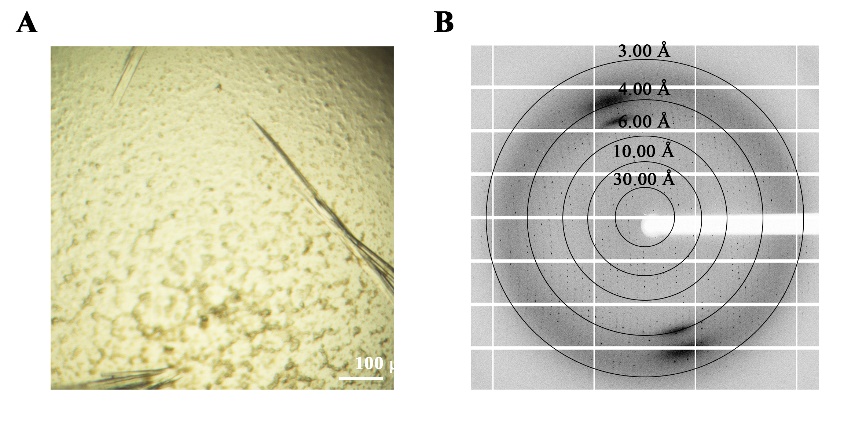


**Figure S2** The complex structure of IgG-Fc and C1q-GR (PDB ID: 6FCZ, left) and **t**he residues on C1q involved in interaction (right). C1q-GR in gray, IgG-Fc in green. The residues involved in IgG-Fc interaction on C1q analysed by chimera displayed as pink sticks.


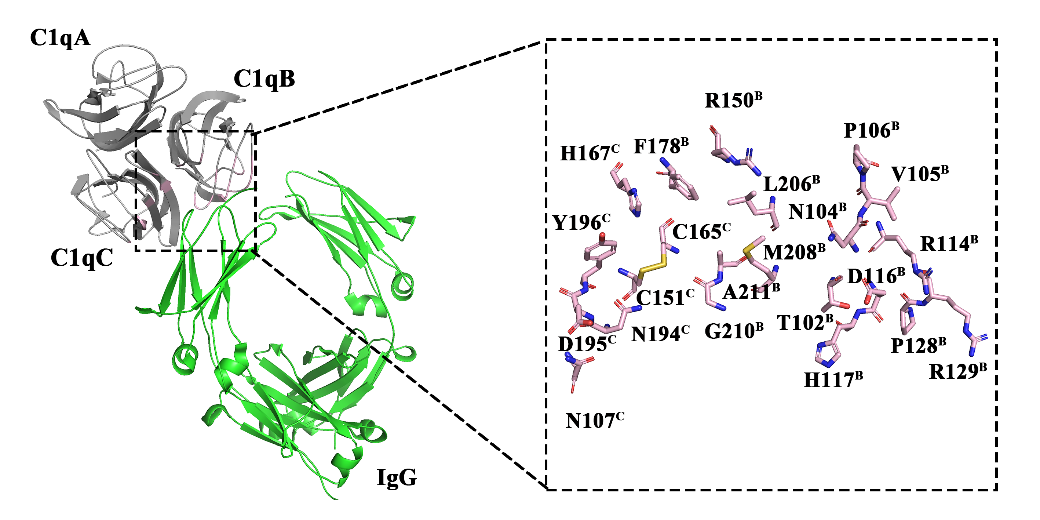


**Figure S3** Sequence alignment between TsCRT and other four helminths CRTs, including *Haemonchus contortus* calreticulin (HcCRT), *Necator americanus* calreticulin (NaCRT), *Brugia malayi* calreticulin (BmCRT), *Opisthorchis viverrini* calreticulin (OvCRT). The eight key residues of TsCRT testified to be involved in C1q interaction were indicated by red triangles.


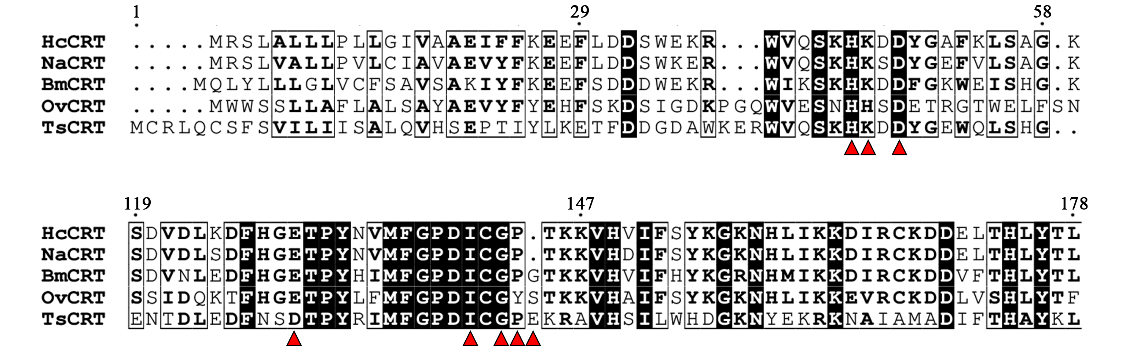

Supplement: Supplementary file 1 [file DataSheet_1.docx]
