# Supplementary material for: Crystal structure of Trichinella spiralis calreticulin and the structural basis of its complement evasion mechanism involving C1q
Source: Front Immunol. 2024 Apr 16;15:1404752. doi: 10.3389/fimmu.2024.1404752 (PMC11059001; doi:10.3389/fimmu.2024.1404752)
Supplement: Supplementary file 2 [file Table_1.docx]

**Table S1** Primers for the construct of TsCRT^Δ^

| **Primer** | **Sequence 5′-3′** |
| --- | --- |
| TsCRT^22–209^ forward (BamHⅠ) | 5′CGGGATCCGAGCCGACCATTTACCTCAAGGAA 3′ |
| TsCRT^22–209^ reverse | 5′TGGTTCAGTCATTGTCCAATCGTCTTC 3′ |
| TsCRT^305–364^ forward | 5′CCAGTACCTGAACTGTATCGATAC 3′ |
| TsCRT^305–364^ reverse (XhoⅠ) | 5′CCGCTCGAGTTTGTCGGATTCCACCTTTTC 3′ |
| TsCRT^Δ^ overlap | 5′ATGACTGAACCAGGTAGTGGTCCAGTACCTGAA 3′ |
